# Effects of Follicular Fluid and Serum Supplementation on Cumulus Cell Expansion and Nuclear Progression of Guinea Pig Oocytes, Using a Baseline Medium Established with Bovine Oocytes

**DOI:** 10.3390/ani15050666

**Published:** 2025-02-25

**Authors:** Jorge X. Samaniego, José L. Pesantez, Luis E. Ayala, Fernando P. Perea, Diego A. Galarza, Jorge B. Dutan, Salvador Ruiz

**Affiliations:** 1 Laboratory of Animal Reproduction Biotechnology, Faculty of Agriculture Sciences, University of Cuenca, Cuenca 010107, Ecuador; jose.pesantez@ucuenca.edu.ec (J.L.P.); luis.ayala@ucuenca.edu.ec (L.E.A.); fernando.perea@ucuenca.edu.ec (F.P.P.); andres.galarza@ucuenca.edu.ec (D.A.G.); jorgeb.dutans@ucuenca.edu.ec (J.B.D.); 2 Department of Physiology, Faculty of Veterinary, University of Murcia, 30100 Murcia, Spain

**Keywords:** guinea pig, in vitro maturation, oocyte, estrous guinea pig follicular fluid (egpFF), estrous guinea pig serum (egpS)

## Abstract

This study evaluated the effects of serum (egpS) and follicular fluid (egpFF) from estrus guinea pigs as in vitro maturation (IVM) supplements for natural cycle guinea pig oocytes. Two experiments were conducted: the first assessed three IVM media, a commercial medium (CMOM) and two homemade media (HMOM-P and HMOM-S), for oocyte in vitro maturation using 615 bovine oocytes, while the second evaluated the incorporation of 5%, 10%, and 20% egpFF or egpS into IVM media for 1744 guinea pig oocytes. Initially, we optimized the IVM base medium using bovine oocytes to determine the most suitable culture conditions. The results obtained from these experiments served as a critical foundation for subsequent supplementation trials conducted with guinea pig oocytes. The oocytes were cultured in 70 μL drops under controlled atmospheric conditions, and maturation rates were assessed based on cumulus cell expansion and nuclear progression. The HMOM-S medium significantly enhanced cumulus cell expansion (72.5 ± 2.88%) compared with the CMOM medium (57.7 ± 5.33%; *p* < 0.05). Supplementation with egpFF at 5% (68.8 ± 6.22%), 10% (76.3 ± 5.39%), and 20% (80.9 ± 6.22%) significantly improved both cumulus cell expansion and nuclear progression in high-quality oocytes (types A and B), compared to the control group (43.3 ± 4.82%). Conversely, supplementation with egpS did not have a significant effect on cumulus cell expansion (*p* > 0.05); however, it notably improved nuclear maturation in low-quality oocytes (type C) at concentrations of 10% and 20% (*p* < 0.05). This resulted in an overall improvement in maturation outcomes, particularly for oocytes with compromised initial quality. These findings demonstrated that the IVM of guinea pig oocytes using HMOM-S medium was significantly enhanced by the presence of egpFF, whereas egpS supplementation exhibited a less pronounced effect on IVM outcomes.

## Simple Summary

This study explores the impact of serum (egpS) and follicular fluid (egpFF) from estrus guinea pigs as supplements on in vitro maturation (IVM) of naturally cycling guinea pig oocytes. The findings suggest that the incorporation of high levels of egpS and egpFF substantially enhances both cumulus cell expansion and nuclear progression of oocytes. While egpFF had the most significant impact, egpS improved nuclear maturation in lower quality oocytes, leading to an overall enhancement in IVM outcomes. Conversely, low-quality oocytes exhibited diminished cell expansion and nuclear progression, irrespective of the egpS and egpFF concentrations employed. These findings hold considerable pertinence for assisted reproductive techniques (ARTs), as they suggest that egpFF supplementation can enhance the efficiency of IVM in guinea pigs. By enhancing oocyte competence, this opens a promising avenue for optimizing assisted reproductive outcomes in this mammalian species. This advancement is pivotal for the formulation of strategies aimed at facilitating guinea pig conservation and reproduction, which could significantly impact the fields of biomedical research and animal production.

## 1. Introduction

Guinea pigs (*Cavia porcellus*) are hystricomorph rodents belonging to the family Caviidae, native to the Andes and domesticated for over 3000 years [1]. These animals are notable not only for their nutritional value [2] and exceptional feed conversion efficiency [3], but also for their importance in biomedical and veterinary research [4]. Their suitability as a model for human reproduction and other higher mammals is well established [5], owing to traits such as their prolonged estrous cycle, contrasting with species such as rats and mice [6], and interstitial placental development with syncytiotrophoblast formation, which closely resembles human physiology [7]. Beyond their role in research, guinea pigs hold substantial economic and nutritional value, particularly in South America, where they are extensively bred for meat production. With the growing demand for guinea pig meat, enhancing reproductive efficiency has become a critical goal for sustainable production systems. However, their reproductive physiology presents significant challenges, including small litter sizes [8], difficulties in estrus synchronization, and a limited ovarian response to superovulation protocols [5,9]. These limitations impede the implementation of conventional breeding techniques, underscoring the necessity of employing assisted reproductive technologies (ARTs) to improve reproductive performance and support the expansion of sustainable production practices. Moreover, information on the optimal conditions for embryo production and development in this species remains scarce [10].

In contrast, for species such as cattle [11], sheep [12], pigs [13], rabbits [14], and mice [15], the processes of maturation, fertilization, and in vitro culture are well standardized and consistently yield reliable results globally. However, in guinea pigs, the in vitro maturation (IVM) rate exhibits significant variability and contradictory outcomes, despite the similarity in the composition of the maturation media used [7,10,16,17]. This inconsistency is likely due to the reduced quality and competence of the female gamete in the guinea pig, which critically affects the success rate of IVM [18,19]. Achieving proper synchrony between nuclear and cytoplasmic maturation of the oocyte is essential for successful IVM [20]. Nuclear maturation involves the resumption of meiosis, a vital process for producing functional gametes. Conversely, cytoplasmic maturation, though not yet fully understood, encompasses substantial organelle reorganizations and the accumulation of specific molecules, such as proteins, lipids, messenger RNA (mRNA), and carbohydrates [21]. These molecules are indispensable for fertilization and the subsequent initiation of embryonic development [22].

A common strategy to enhance the IVM process across various species involves the use of commercial IVM media, which may be defined or semi-defined. These media are formulated to provide essential nutrients and minimize variability, ensuring consistent outcomes across different laboratories [23]. Many of these media are supplemented with reproductive tract-derived fluids, such as follicular fluid [24], fetal serum [25], growth factors [26], hormones [27], and other additives. These supplements play a critical role in enhancing embryonic development and improving the quality of the resulting blastocysts, thereby facilitating the standardization of IVM protocols across various species.

The first report on the IVM of guinea pig oocytes was published by Jagiello in 1969, who observed that 80% of oocytes isolated from female ovaries during the mid-estrous cycle (days 5–8) reached metaphase II after 14 h of culture [28]. However, the composition of the culture medium used was not specified, hindering the replication of these results. In 1974, Yanagimachi described a medium for the IVM of guinea pig oocytes that achieved a 65% maturation rate. This medium consisted of eight parts TCM-199 (Hanks’ base solution) and two parts calf serum, supplemented with NaHCO_3_ (2 mg/mL), K-penicillin G (100 units/mL), and streptomycin sulphate (50 µg/mL) [29]. More recently, studies have utilized a base medium (TCM-199) supplemented with cysteamine (Cys), leukemia inhibitory factor (LIF), and the Rho-associated kinase inhibitor Y27632, reporting a 61.8% maturation rate when Cys and LIF were used in combination [7]. However, these results have been inconsistent and difficult to replicate, underscoring the need for further research to develop a standardized maturation medium that reliably improves the IVM rate of guinea pig oocytes.

It was hypothesized that incorporating follicular fluid or guinea pig serum into standard bovine IVM media could significantly enhance the nuclear maturation of guinea pig oocytes. The addition of these fluids has previously been shown to improve the maturation and developmental potential of oocytes in other species, including bovine [30], equine [31], and porcine [32]. However, to the best of our knowledge, no studies have yet explored the effects of these fluids on the IVM of guinea pig oocytes. Thus, the aim of this study was to evaluate the impact of supplementing IVM media with estrous guinea pig follicular fluid (egpFF) and estrous guinea pig serum (egpS) on the morphological parameters and nuclear progression of guinea pig oocytes.

## 2. Materials and Methods

### 2.1. Chemicals and Media

Unless otherwise indicated, all chemicals required for preparing the experimental media for in vitro maturation (IVM) were purchased from Sigma (St. Louis, MO, USA). Folltropin was obtained from Vetoquinol (Alcobendas, Madrid, Spain), and the commercial culture medium was sourced from IVF-Bioscience (Falmouth, Cornwall, UK).

### 2.2. Experiment 1: Effects of Three In Vitro Maturation Media on Cumulus Cell Expansion and IVM Rate of Bovine Oocytes

The aim of this experiment was to compare the efficiency of three IVM media on cumulus cell expansion and IVM rate to establish a base IVM medium for experiment 2.

#### 2.2.1. Oocyte Collection and Classification

Bovine ovaries were obtained from a local slaughterhouse and transported to the laboratory in saline solution (0.9% NaCl) at 35–37 °C. The ovaries were washed three times, and cumulus–oocyte complexes (COCs) were obtained by aspirating 3 to 8 mm follicles. The COCs were then washed three times in modified Ringer’s lactate containing 1% (*wt*/*v*) of polyvinyl alcohol (PVA). Thereafter, the oocytes were classified according to the criteria described by Hawk and Wall [33] and Ayala et al. [34]. Only COCs with a compact cumulus mass comprising more than three layers were selected for the experiment. After morphological selection, a total of 615 COCs were randomly assigned to experimental groups and control.

#### 2.2.2. In Vitro Maturation (IVM)

In vitro maturation was conducted using a commercial medium (CMOM) and two homemade media for oocyte in vitro maturation (HMOM-P and HMOM-S) (Table 1). The composition of these media is summarized in Table 1, highlighting the differences in supplements and components used in each formulation.

Each group of COCs was washed twice in its respective maturation medium. In vitro maturation was performed in 70 μL drops containing 15 oocytes per drop, using IVM medium (CMOM, HMOM-P, or HMOM-S). The procedure was conducted at 38.8 °C in an atmosphere with 6% CO_2_ at 93% humidity for 24 h.

#### 2.2.3. Cumulus Oocyte Complex Expansion Evaluation

After maturation, cumulus cell expansion was assessed using the subjective scoring proposed by Lorenzo et al. [35]. Briefly, this system categorizes expansion into three increasing degrees: non-expanded cumulus (N-EC), characterized by minimal morphological changes; partially expanded cumulus (P-EC); and totally expanded cumulus (T-EC), where all layers of cumulus cells have expanded, including those closest to the oocyte.

#### 2.2.4. Fixation and Cell Staining

Following maturation, COCs were denuded of cumulus cells and fixed in 0.5% glutaraldehyde in Dulbecco’s Phosphate Buffered Saline (DPBS) for 30 min at room temperature. After fixation, the oocytes were washed and stained with Hoechst 33342 (1 mg/mL) for 15 min in the dark at room temperature. The mounting media consisted of DPBS with glycerol and Hoechst 33342, and the slides were sealed with nail polish. Evaluation was performed using an epifluorescence microscope (Eclipse E200, Nikon, Tokyo, Japan) at 200× and 400× magnifications. Oocytes were considered mature if the first polar body was visible and the metaphase plate was present.

### 2.3. Experiment 2: Effects of Estrous Guinea Pig Serum and Estrous Guinea Pig Follicular Fluid on the IVM Rate

The aim of the second experiment was to evaluate the effects of different percentages of estrous guinea pig serum (egpS) and estrous guinea pig follicular fluid (egpFF) on the IVM rate of guinea pig oocytes.

#### 2.3.1. Estrous Guinea Pig Serum and Follicular Fluid

The egpS and egpFF samples were collected from guinea pigs whose estrous cycle had been synchronized using 0.22 mg/kg Altrenogest (Regumate, MSD Animal Health, Beaucouzé, France) administered for 15 days [5]. The blood and follicular fluid were centrifuged at 2500 rpm for 15 min. The supernatants were aspirated, sequentially filtered through 0.45 μm and 0.22 μm filters, aliquoted, and stored at −20 °C until use. The same batch was used for all experiments. Prior to use, heat inactivation was performed at 56 °C for 30 min.

#### 2.3.2. Guinea Pig Oocyte Collection and Classification

Guinea pig ovaries were collected from specialized slaughter centers and transported to the laboratory in saline solution (0.9% NaCl) at a temperature between 35 and 37 °C. The ovaries were washed three times and COCs were collected by slicing. The oocytes were classified based on morphological criteria described by Wang et al. [7] and Yao et al. [17]. They were categorized into three distinct types according to their cumulus cell structure: type A—oocytes surrounded by four or more layers of cumulus cells; type B—oocytes with one to three layers of cumulus cells; and type C—oocytes that were either denuded or exhibited scattered cumulus cells around the zona pellucida. Oocytes from all three categories were included in the experiments to ensure comprehensive evaluation.

#### 2.3.3. In Vitro Maturation

To assess the effects of different concentrations of egpS and egpFF on the IVM rate, the selected COCs were first washed twice in the HMOM-S medium, which had been identified as the most effective base medium in Experiment 1. This medium was used as the control medium for all experimental treatments in Experiment 2. The HMOM-S medium was formulated based on TCM-199 with Earle’s salts and supplemented with 10% fetal bovine serum (FBS), 0.2 mM sodium pyruvate, 25 μg/mL FSH, 5 μg/mL LH, 30 ng/mL EGF, 50 μg/mL gentamycin sulfate, and 5 μg/mL estradiol-17β. The COCs were then randomly grouped and assigned to the following experimental conditions: control (basic medium), serum groups (basic medium with 5% 10%, or 20% egpS), and follicular fluid groups (basic medium with 5%, 10%, or 20% egpFF). Oocytes of all three types (types A, B, and C) were included in the experiments. A total of 1744 COCs were distributed among eight experimental groups, with three drops (replicates) per group per repetition, corresponding to the three oocyte types. Each drop contained 20 COCs. Manual randomization was performed by sequentially distributing the COCs from each type among the experimental groups to ensure unbiased and balanced allocation. The experiment was conducted with over 12 repetitions. IVM was performed in 50 μL drops at 38.8 °C under an atmosphere of 6% CO_2_ and 93% humidity. After a period of 24 h, the assessment of nuclear maturation (IVM rate) in oocytes was conducted. Oocytes were considered matured when the first polar body was visible, and the metaphase plate was present. These characteristics were identified using Hoechst 33342 fluorescence.

### 2.4. Statistical Analysis

Data were analysed using InfoStat^®^ version 2008 [36]. The Kolmogorov–Smirnov test was applied to assess the normality of numerical data across all variables. Since the data did not follow a normal distribution, percentage data were transformed using an arcsine transformation before conducting parametric statistical analyses. The effects of follicular fluid and serum supplementation on cumulus cell expansion and oocyte maturation rates were evaluated for statistical significance using one-way ANOVA. Differences between groups were analysed using the parametric Di Rienzo, Guzmán, and Casanoves (DGC) test. Data are presented as mean ± standard error of the mean (SEM), with differences considered statistically significant when *p* < 0.05.

## 3. Results

### 3.1. Effects of Three In Vitro Maturation Media on Cumulus Cell Expansion and IVM Rate of Bovine Oocytes

**Figure 1:**
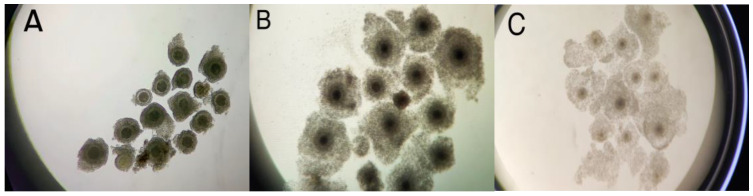
Different degrees of expansion of cumulus cells of bovine oocytes matured in vitro for 24 h. The degree of cumulus cell expansion was classified into three grades: (**A**) non-expanded cumulus (N-EC), (**B**) partially expanded cumulus (P-EC), and (**C**) totally expanded cumulus (T-EC).

Morphological evaluation of bovine oocyte–cumulus complexes revealed varying degrees of cumulus cell expansion, highlighting distinct morphological changes associated with the maturation process, as shown in Figure 1.

**Figure 2:**
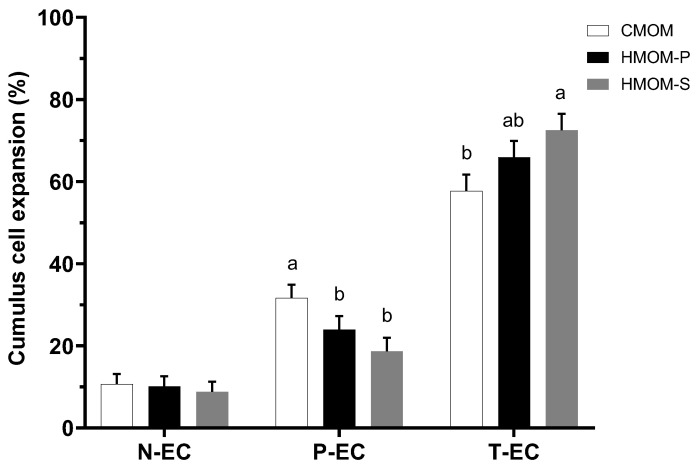
Evaluation of cumulus cell expansion of oocyte bovine based on the classification of the degree of expansion achieved 24 h after treatment with three in vitro maturation media: CMOM (Commercial Medium for Oocyte Maturation), HMOM-P (Homemade Medium for Oocyte Maturation—Primary), and HMOM-S (Homemade Medium for Oocyte Maturation—Secondary). The degree of cumulus cell expansion was classified according to three grades: N-EC (non-expanded cumulus), P-EC (partially expanded cumulus), and T-EC (totally expanded cumulus). Different lowercase letters (a, b) indicate significant differences between treatment for each degree of expansion of cumulus cells (*p* < 0.05).

A total of 615 bovine oocytes were subjected to in vitro maturation, and the degree of cumulus cell expansion was evaluated after 24 h of culture, as shown in Figure 2. The results demonstrate that HMOM-S significantly increased the proportion of totally expanded cumulus compared to the commercial oocyte maturation medium (CMOM), reaching 72.5% vs. 57.7% (*p* < 0.05). However, no significant difference was observed between HMOM-S and HMOM-P (Figure 2). When assessing the partially expanded cumulus, both homemade media, HMOM-P (23.9%) and HMOM-S (18.7%), exhibited lower and comparable cumulus cell expansion rates (*p* > 0.05) compared to CMOM, which showed a significantly higher percentage of partially expanded cumulus (31.66%; *p* < 0.05; Figure 2). Conversely, the three treatments HMOM-P (10.1%), HMOM-S (8.8%), and CMOM (10.7%), showed comparable percentages of COCs with minimal or no expansion (N-EC; Figure 2), with no statistically significant differences among groups (*p* > 0.05). Overall, these results highlight the efficacy of the HMOM-P and HMOM-S treatments promoting cumulus cell expansion, demonstrating a remarkable effectiveness compared to the commercial medium.

**Figure 3:**
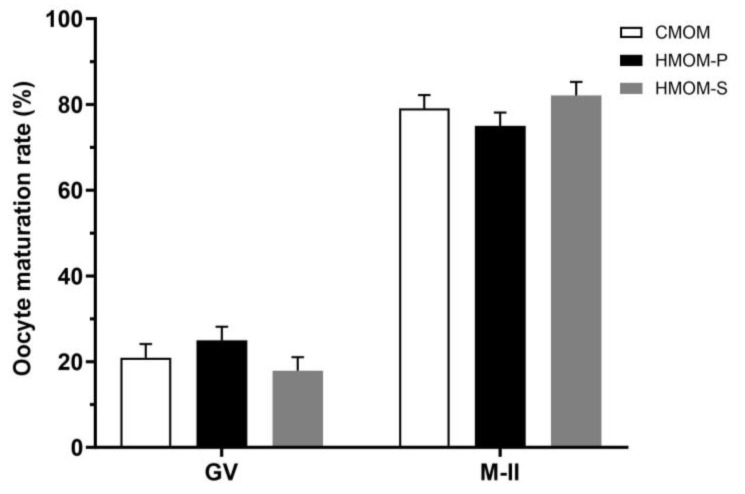
Evaluation of bovine oocyte in vitro maturation rates based on the progression from the germinal vesicle stage (GV) to metaphase II (M-II), assessed 24 h post-treatment with three distinct in vitro maturation media: CMOM (Commercial Medium for Oocyte Maturation), HMOM-P (Homemade Medium for Oocyte Maturation—Primary), and HMOM-S (Homemade Medium for Oocyte Maturation—Secondary).

In the assessment of IVM rates of COCs reaching metaphase II (M-II), as indicated by the presence of a polar body and metaphase plate, all treatments, HMOM-P (74.9%), HMOM-S (82.2%), and CMOM (79.0%) exhibited similar and higher IVM rates (Figure 3; *p* > 0.05) after 24 h of culture.

These results suggest that, despite variations in percentages, all media tested are effective in supporting progression to M-II. Furthermore, it is important to note that, although oocytes may appear morphologically competent, not all have the capacity to reach M-II. This suggests that morphology alone is not a reliable indicator of oocyte competence to complete the maturation process.

### 3.2. Experiment 2: Effects of Serum and Follicular Fluid from Estrous Guinea Pigs on Cumulus Cell Expansion and IVM Rate of Guinea Pig Oocytes

Based on the results from the bovine oocyte experiment, the medium identified as optimal for in vitro maturation was selected as the base medium for guinea pig oocyte maturation. The effects of different concentrations of egpFF (5%, 10%, and 20%) on cumulus cell expansion and nuclear maturation rates, based on morphological classification of natural cycle guinea pig oocytes, are presented in Table 2 and Table 3.

In the control group, no significant differences were observed among the three oocyte types (Table 2). However, when egpFF was added to the basic medium, positive effects on cumulus cell expansion were observed (*p* < 0.05). The addition of 5% egpFF enhanced the expansion of type A and B oocytes compared to type C oocytes, which did not show a notable response to the treatment. However, the most pronounced effects on cumulus cell expansion in type A oocytes were observed with 5%, 10%, and 20% egpFF (Table 2). Regarding type B oocytes, a higher response was observed with 10% and 20% egpFF. These findings suggest that higher concentrations of egpFF promote greater cumulus cell expansion, especially in higher quality oocytes such as types A and B (*p* < 0.05).

As shown in Table 3, no significant differences were observed among oocyte types within the control group (*p* > 0.05). However, when egpFF concentrations of 5%, 10%, and 20% were added to the basic medium, there was a significant increase in the proportion of quality A oocytes reaching M-II. Nevertheless, the addition of a high concentration of egpFF (10% or 20%) did not show a significant effect on this oocyte type (*p* > 0.05).

In this experiment, we also evaluated the effects of different concentrations of egpS (5%, 10%, and 20%) on cumulus cell expansion (Table 4) and nuclear maturation rate (Table 5), based on the morphological classification of natural cycle guinea pig oocytes.

Treatments with different egpS concentrations resulted in similar rates of cumulus cell expansion across A-, B-, and C-quality oocytes (*p* > 0.05). However, when compared within the same treatment, significant differences were observed, with greater rates of cell expansion in higher quality A and B oocytes compared to C-quality oocytes, which exhibited significantly lower expansion rates (*p* < 0.05).

The rate of nuclear maturation was similar among guinea pig oocytes of types A, B, and C within the same treatment group (*p* > 0.05). However, type A and B oocytes, considered the highest quality oocytes, exhibited significantly higher and similar nuclear maturation rates compared to the control group (*p* < 0.05). In contrast, type C oocytes showed a less pronounced but significant response, particularly at the higher concentrations of 10% and 20%. These findings suggest that type A and B oocytes are more sensitive to estrus guinea pig serum, as evidenced by their enhanced response across all concentrations.

## 4. Discussion

This experiment was first conducted in bovine oocytes to evaluate the efficacy of three in vitro maturation media, allowing us to establish an optimal baseline before applying the selected medium to guinea pig oocytes. This analysis is essential for establishing a reliable baseline IVM medium that not only supports the IVM medium conditions used in Experiment 2 but also serves as a valuable reference for future research in this field.

The results demonstrate that the percentage of total cumulus cell expansion (T-EC) was significantly higher in the HMOM-S medium compared to the CMOM medium (*p* < 0.05) (Figure 2). This finding suggests that the inclusion of epidermal growth factor (EGF, 30 ng/mL) in the HMOM-S medium, likely absent in the CMOM medium, could play a critical role in enhancing cell expansion. EGF is well documented for promoting cumulus cell proliferation and differentiation, primarily through the activation of signaling pathways such as MAPK (Raf/mitogen-activated protein kinase) and AKT (phosphatidylinositol 3-kinase [PI3K]/protein kinase B),which are essential for oocyte growth and maturation [37,38]. Previous studies have demonstrated that EGF supplementation improves oocyte competence and accelerates maturation by activating key metabolic processes [10]. For instance, the addition of 50 ng/mL EGF to the IVM base medium resulted in a 75.9% increase in oocytes progressing to metaphase II, compared to 43.5% in the control group [39].

Furthermore, in this experiment, the homemade maturation media (HMOM-P and HMOM-S) demonstrated comparable total expansion percentages (T-EC) (*p* > 0.05), suggesting that the addition of fetal bovine serum (FBS) exerted a balancing effect on the outcomes. Cumulus expansion plays a critical role in various reproductive events, including the completion of meiosis, ovulation, the capture of the cumulus–oocyte complex by the oviduct, fertilization, and early embryonic development [40].

Fetal bovine serum is a natural medium rich in proteins, enzymes, hormones, growth factors, cytokines, fatty acids, carbohydrates, and vitamins [41]. These components not only facilitate reproductive events but are also essential for cell growth [42], proliferation [43], and stabilization of the extracellular matrix of the cumulus cells. Specifically, glycoproteins such as pre-α-trypsin inhibitor (PαI) in conjunction with hyaluronic acid (HA) help maintain the integrity of this matrix by preventing its collapse and ensuring an optimal environment for oocyte development [44,45].

One of the key characteristics of competent oocytes is their ability to resume meiosis I and progress to metaphase II [21]. This resumption is critical for completing nuclear maturation, a key process that prepares the oocyte for successful fertilization. In Experiment 1, we assessed the IVM rate of bovine oocytes after exposure to three maturation media: CMOM (79%), HMOM-P (74.9%), and HMOM-S (82.1%). Although these percentages were high in this study, no significant statistical differences (*p* > 0.05) were observed among treatments (Figure 3). In addition, fewer than 20% of the COCs in the three treatments failed to resume meiosis and remained at the germinal vesicle stage. These findings align with previous studies that have reported similarly high percentages [35,46,47] of nuclear maturation for this species using comparable IVM media.

These percentages suggest that the homemade maturation media (HMOM-P and HMOM-S) promote cell and nuclear maturation yields comparable to commercial media used in various studies [48,49].

The efficiency of the in vitro maturation (IVM) process is crucial for the success of in vitro fertilization (IVF) and in vitro culture (IVC) across various species. In the case of guinea pig oocyte IVM, variable success rates have been documented with the use of media supplements [7,10,16,17,29,39]. This study is the first to explore the use of follicular fluid (egpFF) and serum (egpS) from estrous guinea pigs as supplements in a base medium originally developed and validated for cattle.

The results indicate that oocytes of type A matured in the medium supplemented with 20% egpFF showed a 1.9-fold increase in cumulus cell expansion compared to the control treatment and a 1.2-fold increase compared to the medium supplemented with 5% egpFF. However, supplementation with 10% or 20% egpFF yielded a similar percentage expansion.

The observed effects of egpFF supplementation on cumulus cell expansion and nuclear maturation in guinea pig oocytes are likely mediated by the presence of bioactive molecules within the follicular fluid, such as growth factors (e.g., EGF and IGF), steroids, glycosaminoglycans, and other signaling molecules. These bioactive components are essential for activating key intracellular pathways, including MAPK and PI3K/AKT, which play pivotal roles in cumulus cell function, oocyte development, and the overall maturation process [37,38,50].

The interaction between cumulus cells and oocytes via gap junctions further facilitates the transfer of ions, metabolites, and regulatory signals, enhancing the efficiency of maturation [40,51]. The results of this study suggest that supplementation with egpFF contributes to these molecular pathways, thereby improving the maturation outcomes observed, particularly in oocytes of type A, where significant increases in cumulus cell expansion were noted. This aligns with the idea that follicular fluid components are critical for optimizing oocyte maturation in guinea pigs.

Interestingly, the differential responses observed between oocyte types A, B, and C may reflect inherent differences in their ability to utilize the bioactive components of egpFF. Type A oocytes, with a robust cumulus cell structure, are better equipped to mediate nutrients and signal transfer, which is likely to explain their enhanced response to egpFF supplementation. In contrast, types B and C, which lack this structural advantage, may struggle to benefit from the bioactive molecules present in egpFF, leading to a less pronounced effect. These observations further emphasize the critical role of cumulus cells in facilitating oocyte competence during IVM and highlight the need to consider oocyte type when evaluating maturation outcomes. This function of COCs is mediated by intercellular communication involving gap junctions and signal transduction pathways, highlighting the importance of cumulus cells in the maturation process.

In addition, oocyte growth and development depend on the supply of nutrients from surrounding follicular cells [51]. The viability and steroidogenic capacity of cumulus cells are critical for achieving oocyte competence during IVM [40]. Our findings align with studies in other species, such as cattle and pigs, where low-dose follicular fluid supplementation enhanced cumulus cell expansion, increased fertilization rates, and promoted embryo development [51,52].

However, some studies highlight that the variable nature of follicular fluid may lead to inconsistent and sometimes contradictory results [53,54]. This variability may be attributed to differences in the physiological state of the donors, hormonal fluctuations, and individual metabolic conditions, which could impact the composition and effectiveness of FF supplementation in IVM. Despite these challenges, egpFF supplementation remains a promising strategy for improving IVM outcomes in understudied species such as the guinea pig. To optimize its use, further studies are needed to characterize the molecular composition of guinea pig follicular fluid and its interactions with various oocyte types. Such research could reveal species-specific differences and help optimize the use of follicular fluid as a supplement in IVM systems. Moreover, a deeper understanding of these molecular mechanisms would not only improve IVM outcomes but also contribute to advancing reproductive technologies for guinea pigs, a species with significant potential for research and conservation.

Nuclear maturation rates were observed to be considerably lower compared to the extent of cumulus cell expansion. In the control treatment, only 45% of oocytes of type A reached metaphase II, a percentage notably lower than that of COCs exhibiting cell expansion (see Table 2). This discrepancy has practical implications, as the asynchrony between morphological and nuclear maturation may impair the selection of suitable oocytes for in vitro fertilization, potentially resulting in lower yields [55].

However, supplementation with egpFF at concentrations of 10% and 20% effectively mitigated this discrepancy. Although nuclear maturation decreased in the egpFF 20% group, the reduction was less pronounced than in the control (no egpFF), with percentages of 32.7% and 35.5%, respectively (see Table 3). These results suggest that the interaction between type A COCs and egpFF supplementation at concentrations of 10% and 20% positively influenced nuclear maturation compared to the control and lower egpFF concentration (egpFF 5%).

In contrast, type B and C COCs did not exhibit significant effects in nuclear maturation in any of the treatments. This outcome may be attributed to limited intercellular communication and the reduced number of cumulus cells in these COC types, both of which are essential for optimal oocyte maturation. These findings align with previous studies showing improved nuclear maturation rates in type A COCs in media enriched with hormonal factors and amino acids, highlighting the critical role of these components in the in vitro maturation process for this species [17].

Finally, the addition of estrus guinea pig serum (egpS) demonstrated a positive effect on cumulus cell expansion, suggesting that egpS promotes this process. However, in the case of low-quality oocytes (type C), egpS supplementation did not affect cumulus cell expansion (*p* > 0.05). This indicates that the efficacy of such supplements depends on the oocyte type, underscoring the importance of considering the quality of these structures when assessing the impact of additives on IVM processes.

Regarding nuclear maturation, a 3.7- and a 2.9-fold increase were observed in type A and B oocytes, respectively, compared to the control group (Table 5). However, the percentages of nuclear maturation observed in this study were lower than those reported in a recent study [10]. This suggests that, although serum may affect cumulus cells in this species, cumulus cell expansion cannot be considered a reliable indicator of nuclear maturation, unlike findings in other species [21,56].

These results highlight the need for further investigation into the relationship between cumulus cell expansion and nuclear maturation in this species. Notably, in guinea pigs, in vitro maturation is a complex process that requires a comprehensive understanding of the cellular interactions and environmental factors involved.

Given the complexity, future studies should focus on optimizing experimental conditions, exploring the interaction between various media and supplements, and improving nuclear maturation rates to advance reproductive technologies for this species. These findings provide a foundation for developing in vitro production (IVP) systems for guinea pig embryos, which could enhance reproductive efficiency and conservation efforts for this species.

## 5. Conclusions

The IVM protocol proposed in this study was effective in promoting cumulus cell expansion and oocyte nuclear maturation in guinea pigs. The inclusion of estrus guinea pig follicular fluid (egpFF) enhanced both processes. In addition, estrus guinea pig serum (egpS) contributed to cumulus cell expansion, although its efficacy was limited in lower quality oocytes. These results contribute to the development of more efficient in vitro maturation (IVM) protocols, which could serve as a crucial step toward establishing in vitro production systems (IVP) for guinea pig embryos.

## Figures and Tables

**Table 1:** Comparison of in vitro maturation media used in the study.

Component	CMOM (Commercial)	HMOM-P	HMOM-S
Base medium	Not specified	TCM-199 with Earle’s salts	TCM-199 with Earle’s salts
FBS (10%)	Not specified	✓	✓
Sodium pyruvate (0.2 mM)	Not specified	✓	✓
FSH (25 μg/mL)	Not specified	✓	✓
LH (5 μg/mL)	Not specified	✓	✓
Estradiol-17β (5 μg/mL)	Not specified	✓	✓
Gentamicin (50 μg/mL)	Not specified	✓	✓
L-glutamine (2 mM)	Not specified	✓	✗
Cysteamine (100 μM)	Not specified	✓	✗
EGF (30 ng/mL)	Not specified	✗	✓

Abbreviations: FBS (fetal bovine serum), EGF (epidermal growth factor), FSH (follicle-stimulating hormone), LH (luteinizing hormone). A check mark (✓) indicates the presence of the component, while a cross (✗) indicates its absence. CMOM refers to the commercial maturation medium, while HMOM-P and HMOM-S are homemade maturation media based on TCM-199 with Earle’s salts.

**Table 2:** Effects of different concentrations of egpFF on cumulus cell expansion of oocytes collected from natural cycling guinea pigs.

Oocyte Type	Control (no egpFF)	egpFF 5%	egpFF 10%	egpFF 20%
A	43.3 ± 4.82 ^b^	68.8 ± 6.22 ^A; a^	76.3 ± 5.39 ^A; a^	80.9 ± 6.22 ^A; a^
B	43.5 ± 4.82 ^b^	55.1 ± 6.22 ^A; b^	70.6 ± 5.39 ^A; a^	65.9 ± 6.22 ^A; a^
C	42.9 ± 4.82	46.7 ± 6.22 ^B^	44.1 ± 5.39 ^B^	53.1 ± 6.22 ^B^

Mean (± SEM) cumulus cell expansion rates of types A, B, and C oocytes cultured under different concentrations (5%, 10%, and 20%) of egpFF (estrous guinea pig follicular fluid). The control (no egpFF) group refers to oocytes cultured without the addition of egpFF. Different uppercase letters (^A^, ^B^) within the same column indicate significant differences between oocyte type within the same treatment, while different lowercase letters (^a^, ^b^) within the same row denote significant differences between treatments within the same oocyte type (*p* < 0.05).

**Table 3:** Effects of different concentrations of egpFF on nuclear in vitro maturation rate of oocytes collected from natural cycling guinea pigs.

Oocyte Type	Control (no egpFF)	egpFF 5%	egpFF 10%	egpFF 20%
A	23.8 ± 3.14 ^b^	41.8 ± 3.51 ^A; a^	51.4 ± 3.51 ^A; a^	52.2 ± 4.06 ^A; a^
B	14.1 ± 3.14	24.3 ± 4.06 ^B^	27.0 ± 3.51 ^B^	29.4 ± 4.06 ^B^
C	13.4 ± 3.14	12.1 ± 4.06 ^B^	17.3 ± 3.51 ^B^	18.7 ± 4.97 ^B^

Mean (± SEM) IVM rates of types A, B, and C oocytes cultured under different concentrations (5%, 10%, and 20%) of egpFF (estrous guinea pig follicular fluid). The control (no egpFF) group refers to oocytes cultured without the addition of egpFF. Different uppercase letters (^A^, ^B^) within the same column indicate significant differences between oocyte type within the same treatment, while different lowercase letters (^a^, ^b^) within the same row denote significant differences between treatments within the same oocyte type (*p* < 0.05).

**Table 4:** Effects of different concentrations of egpS on cumulus cell expansion of oocytes collected from natural cycling guinea pigs.

Oocyte Type	Control (no egpS)	egpS 5%	egpS 10%	egpS 20%
A	69.7 ± 3.63 ^A^	84.7 ± 3.98 ^A^	84.5 ± 3.63 ^A^	87.4 ± 3.98 ^A^
B	65.9 ± 3.63 ^A^	85.8 ± 3.98 ^A^	81.9 ± 3.63 ^A^	86.4 ± 3.98 ^A^
C	40.1 ± 3.36 ^B^	38.9 ± 3.36 ^B^	39.8 ± 3.63 ^B^	34.0 ± 3.98 ^B^

Mean (± SEM) cumulus cell expansion rates of types A, B, and C oocytes cultured under different concentrations (5%, 10%, and 20%) of egpS (estrous guinea pig serum). The control (no egpS) group refers to oocytes cultured without the addition of egpS. Different uppercase letters (^A^, ^B^) within the same column indicate significant differences between oocyte type within the same treatment.

**Table 5:** Effects of different concentrations of egpS on nuclear in vitro maturation rate of oocytes collected from natural cycling guinea pigs.

Oocyte Type	Control (no egpS)	egpS 5%	egpS 10%	egpS 20%
A	8.7 ± 2.79 ^b^	25.9 ± 3.06 ^A; a^	26.9 ± 2.79 ^a^	32.5 ± 3.06 ^a^
B	10.0 ± 2.79 ^b^	25.7 ± 3.06 ^A; a^	25.7 ± 2.79 ^a^	29.1 ± 3.06 ^a^
C	6.5 ± 2.79 ^b^	10.2 ± 2.79 ^B; b^	18.0 ± 2.79 ^a^	21.5 ± 3.06 ^a^

Mean (± SEM) IVM rates of types A, B, and C oocytes cultured under different concentrations (5%, 10%, and 20%) of egpS (estrous guinea pig serum). The control (no egpS) group refers to oocytes cultured without the addition of egpS. Different uppercase letters (^A^, ^B^) within the same column indicate significant differences between oocyte type within the same treatment, while different lowercase letters (^a^, ^b^) within the same row denote significant differences between treatments within the same oocyte type (*p* < 0.05).

## Data Availability

The data that support the findings of this study are available from the corresponding author upon reasonable request.
